# Levetiracetam Versus Levetiracetam Plus Sodium Channel Blockers for Postoperative Epileptic Seizure Prevention in Brain Tumor Patients

**DOI:** 10.7759/cureus.24894

**Published:** 2022-05-10

**Authors:** Noriyuki Watanabe, Eiichi Ishikawa, Narushi Sugii, Kazuki Sakakura, Masahide Matsuda, Hidehiro Kohzuki, Takao Tsurubuchi, Yosuke Masuda, Alexander Zaboronok, Hiroyoshi Kino, Mikito Hayakawa, Shingo Takano, Yuji Matsumaru, Hiroyoshi Akutsu

**Affiliations:** 1 Department of Neurosurgery, Faculty of Medicine, University of Tsukuba, Tsukuba, JPN; 2 Epileptic Center, University of Tsukuba Hospital, Tsukuba, JPN

**Keywords:** early seizures, brain tumor, postoperative management, anti-epileptic drug, non-convulsive status epilepticus

## Abstract

Background

Brain tumor patients tend to develop postoperative epileptic seizures, which can lead to an unfavorable outcome. Although the incidence of postoperative epileptic seizures and adverse events are improved with the advent of levetiracetam (LEV), postoperative epilepsy occurs at a frequency of 4.6% or higher. In brain tumor patients, the addition of sodium channel blockers (SCBs) to LEV significantly reduces seizures, though confirmed in a non-postoperative study. Thus, the combination of SCBs with LEV might be promising.

Objective

In this prospective randomized controlled trial we investigated the safety, evaluated by adverse events during one and two weeks after surgery, and the efficacy, evaluated by the incidence of early epilepsy, including non-convulsive status epilepticus (NCSE), of using LEV alone or SCBs added to LEV in patients who underwent craniotomy or biopsy for brain tumors or brain mass lesions.

Methods

Patients with brain tumors or brain mass lesions undergoing surgical interventions, excluding endoscopic endonasal surgery (EES), with a diagnosis of epilepsy were eligible for this study. Patients are randomized into either Group A or B (B1 or B2) after the informed consents are taken; LEV alone in Group A patients, while LEV and SCBs in Group B patients (GroupB1, intravenous fosphenytoin plus oral lacosamide (LCM) and GroupB2, intravenous LCM plus oral LCM) were administered postoperatively. Fifty-three patients were enrolled during the first two and a half years of the study and four of them were excluded, resulting in the accumulation of 49 patients’ data.

Results

Postoperative epileptic seizures occurred only in three out of 49 patients during the first week (6.1%) and in seven patients within two weeks after surgery (14.3%, including the three patients during the first week). In Group A, epileptic seizures occurred in two out of 26 patients during the first week (7.7%) and in five patients within two weeks (19.2%) after surgery. In Group B, epileptic seizures occurred in one out of 23 patients during the first week (4.3%) and in two patients during the first two weeks (8.7%). Low complication grade of epileptic seizures was observed in Group B rather than in Group A, however, without significant difference (p=0.256). There was no difference in the frequency of adverse effects in each group.

Conclusion

Although not statistically significant, the incidence of epileptic seizures within one week after surgery was lesser in LEV+SCBs groups than in LEV alone. No hepatic damage or renal function worsening occurred with the addition of LCM, suggesting the safety of LEV+SCBs therapy.

## Introduction

Postoperative seizures within the first week after surgery are called acute symptomatic seizures or early postoperative seizures (EPSs) and are not necessarily regarded as epilepsy, while late seizures one week after surgery are regarded as true epilepsy, obtaining epileptic lesions [1]. Early seizures often cause can worse consciousness, preventing the assessment of the neurological symptoms during postoperative periods, and may result in a worse outcome. The percentage of early seizures varies depending on the lesions, ranging from 4.9~16%; 15％ in meningioma, 16% in brain metastasis, and 15% glioma [2-4]. In the study with glioma patients involved, factors associated with postoperative seizures included intraparenchymal brain tumors, young age (<50 years), frontal lobe tumor location, and multiple lesions. Among gliomas, World Health Organization (WHO) grade II oligodendrogliomas (ODs) and low-grade tumors are more related to seizures [5]. Non-convulsive status epilepticus (NCSE) is not associated with any convulsions, which often delays the diagnosis and affects the patient's neurological function and is related to a poorer prognosis [6]. Our previous retrospective study showed that the patients with brain tumors in the postoperative phase often developed NCSE [7], which caused difficulty in neurological assessment after surgeries.

Intravenous AEDs are preferably selected both for the prevention of EPS and postoperative epileptic seizures (with the preoperative episode of epilepsy) in the perioperative periods for brain tumor patients. In Japan, fosphenytoin (fPHT), a prodrug of phenytoin (PHT) that is an ordinary sodium channel blocker (SCB), has been used for brain tumor patients, especially for EPS prevention; however, fPHT is associated with side events and potential drug interaction [8]. Levetiracetam (LEV) has also been available as an intravenous drug for brain tumor patients with at least one episode of epilepsy In the previous survey including 1400 patients who underwent surgical invasion at our hospital from January 2015 to August 2017, 388 patients underwent surgery for brain tumors or brain non-tumoral mass lesions, excluding 186 patients who underwent EES for pituitary tumors. Of those, 19 (4.9%) were diagnosed with or suspected of having postoperative epilepsy [7]. In that sub-analysis, the incidence of seizures in patients postoperatively taking LEV was 4.8%, which was lower than the 5.3% incidence in patients postoperatively taking fPHT. And a meta-analysis revealed that LEV was more effective than PHT in terms of epilepsy prevention, and LEV also tended to be better in terms of severe adverse events [9]. Although the incidence of postoperative epilepsy and adverse events seem to be improving with the advent of LEV, both in the abovementioned article and in our previous retrospective analysis [7], postoperative epilepsy occurred with a frequency of approximately 5% or higher, which is not negligible considering it as a postoperative complication.

Lacosamide (LCM) is a novel SCB, already approved in Japan as an oral drug and available as an intravenous drug from 2019. It promotes slow inactivation of voltage-gated sodium channels, and unlike PHT and other drugs, it does not induce or inhibit cytochrome P450, making it easy to use with little interaction with other drugs. According to the VITOBA study, the addition of LCM to patients with partial-onset seizures (regardless of the primary disease) resulted in 45.5% seizure-free cases and 72.5% seizure reduction by 50% or more in three months. This study also shows an add-on effect to LEV (71.2% of patients had a seizure reduction of 50% or more) [10]. In a study limited to brain tumors, the additional prescription of LCM significantly reduced partial seizures, including generalized seizures during the subsequent several months [11]. Thus, the combination of SCBs with LEV might be promising, but there are still no prospective studies investigating the add-on effect of SCBs on the prevention of early postoperative epileptic seizures. 

Therefore, we aimed to investigate the incidence of postoperative epileptic seizures after brain tumor surgery in patients taking LEV alone or LEV plus SCBs. In this prospective randomized controlled trial, we evaluated the treatment safety by incidence and character of adverse events within one and two weeks after surgery, and the treatment efficacy by the incidence of postoperative epilepsy (including NCSE). This was a prospective, randomized, controlled, Phase IIb study to explore the safety and feasibility of proceeding to a Phase III study.

## Materials and methods

Patients

The current study has been performed in accordance with the Declaration of Helsinki. The study protocol was approved by the ethics committee of our institution, the University of Tsukuba Hospital (number H29-221). Informed consent was obtained from all the patients included in the study. Patients with brain tumors or brain mass lesions undergoing surgical interventions, excluding endoscopic endonasal surgery (EES), with a history of epilepsy were eligible for this study. Patients were stratified by seizure control (good/poor) and tumor location (intra-axial/extra-axial). Patients who had been taking AEDs with a provisional diagnosis of epilepsy without seizures in the past. Patients who met all of the following inclusion criteria and none of the exclusion criteria were eligible for enrollment in this study: 1) patients with brain tumors (brain mass lesions) who were scheduled to undergo surgical intervention excluding EES; 2) patients with a history of epilepsy (including those taking AEDs without obvious seizures in the past); 3) patients who were scheduled to receive AEDs after surgery; 4) patients aged 16 years or older at the time of obtaining the consent; 5) patients who had received sufficient explanation and understood it before participating in this study, and who had given written consent by their own free will or that of their guardian.

Patients with any of the following conditions were not included in the study: 1) patients with a history of serious adverse reactions to LEV, PHT, fPHT, or LCM; 2) patients receiving intravenous LEV, fPHT, or LCM (excluding oral medications) before surgery; 3) patients who were taking PHT or LCM preoperatively; 4) patients with poorly controlled diabetes mellitus, a history of myocardial infarction, or unstable angina pectoris; 5) patients with severe liver disease (aspartate amino-transaminase (AST, glutamic-oxaloacetic transaminase [GOT]) or alanine amino-transaminase (ALT, glutamic pyruvic transaminase [GPT]) of 100 U or higher); 6) patients with severe renal disease (serum creatinine ≥ 2.0 mg/dL); 7) patients with diseases that cause consciousness disturbance/disorders or non-epileptic convulsions, such as severe hyponatremia (<130 mg/dL); 8) women who were pregnant, of childbearing potential or breast-feeding; 9) patients who had participated in any other study or received an investigational drug within 3 months before the start of drug administration in the current study, or who were not eligible for this study for any other reason as judged by the investigator.

Study design

According to the metanalysis, postoperative epileptic seizures occurred in 7.69% of brain tumor patients receiving LEV [12]. However, reporting NCSE in 73.7% of all observed seizures implied the possibility of having undetected NCSE in our previous studies [7]. If NCSE were accurately assessed, the incidence of epilepsy in the LEV monotherapy group would be estimated to be up to 29.2% in the prospective study. The feasibility of continuing the study had to be assessed three years after the enrollment; however, only 53 patients were enrolled during the first two and a half years of the study and four of them were excluded (two patients had postoperative seizures just after surgery before the first intravenous administration of AEDs, one patient received oral LCM before surgery and one patient withdrew his informed consent before the first intravenous administration), resulting in the accumulation of 49 patients’ data. Overall, epileptic seizures occurred in seven of 49 patients (14.3%). Considering the expected ratio in each group, it was thought that approximately 100 more cases would be necessary to obtain the significant difference, and therefore, it was decided to discontinue the study and analyze the accumulated data.

Clinical data collection

The presence or absence of postoperative epileptic seizures, hepatic damage estimated by AST and ALT, renal function or damage estimated by creatinine (Cre), estimated glomerular filtration rate (eGFR), other tissue damage estimated by lactate dehydrogenase (LDH), or other adverse events were determined on postoperative days 7 ± 2 and 14 ± 2. The postoperative epileptic seizures were categorized into generalized tonic-clonic seizures (GTCS), NCSE, focal impaired awareness seizures (FIAS), and focal awareness seizures (FAS). In the limited cases, electroencephalogram (EEG) was performed and the diagnosis of NCSE was made based on Salzburg Consensus Criteria [13, 14]. The Clavien-Dindo classification (CDC) was used for postoperative seizures or interventional complications [15]. Grade I was assigned in case of any deviation from the normal postoperative course without the need for pharmacological treatment or surgical, endoscopic, or radiological interventions (allowed therapeutic regimens were drugs such as antiemetics, antipyretics, analgesics, diuretics and electrolytes, and physiotherapy; this grade also includes wound infections developed at the bedside). Grade II was assigned if pharmacological treatment was needed using drugs other than those allowed for grade I complications, including blood transfusion and total parenteral nutrition. Grade III was assigned when surgical, endoscopic, or radiological intervention was required. Grade IVa was assigned in case of a single organ (including CNS complications) life-threatening complication requiring IC/ICU management. For adverse effects such as other postoperative events and abnormal blood test findings, Common Terminology Criteria for Adverse Events (CTCAE) Version 5.0 was used.

Medication administration

In Group A patients (LEV alone), LEV was administered intravenously on the operation day and the next day. In the evening on the day after surgery, or as soon as oral or nasogastric tube administration was feasible, medications were switched to oral tablets. In Group B patients, LEV and LCM had to be administered intravenously on the day of surgery and the next day. LCM as an intravenous (IV) infusion was not approved until the middle of the study period. Therefore, the use of fPHT as an alternative was allowed (Group B1) until the intravenous infusion of LCM became available (Group B2) since the primary anticonvulsant mechanism of these AEDs is sodium channel modulation/ inactivation [16]. The first dose of fPHT was 15-18 mg/kg, and the next dose was 5-7.5 mg/kg. Medications were switched to oral tablets in the evening on the day after surgery or when oral or nasogastric tube administration was available. As for the dosage, LEV had to be 1000 mg/day in principle, and if the preoperative dose was 1250 mg or over, it had to be the same after the surgery. The dose of LCM had to be started with 100 mg/day and could be increased at intervals of one week or more to a maintenance dose of 200 mg/day at the doctor’s discretion, all of which had to be administered divided into two doses daily.

Statistical analysis

Fisher’s exact tests were performed for sex, preoperative seizure control (good or poor), tumor location (intra-axial or extra-axial), and postoperative seizures. Either Student’s t-test or Mann-Whitney test were applied for age and blood test data. Statistical analysis was performed with SPSS version 27 (IBM Corp, Armonk, USA), and p-values < 0.05 were considered to show statistical significance.

## Results

Forty-nine patients were enrolled in this study. In terms of patient background, there were no significant differences in all parameters (Table 1).

**Table 1:** Patient background in Group A who received LEV intravenously after surgery followed by oral LEV administration and in Group B who received LEV and SCBs intravenously followed by oral LEV plus LCM administration. ALT, alanine aminotransaminase; AST, aspartate aminotransaminase; Cre, creatinine; eGFR, estimated glomerular filtration rate; LDH, lactate dehydrogenase; SCBs, sodium channel blockers; CI, confidence interval I, Student’s t-test; II, Fisher’s exact test; III, Mann-Whitney test.

	A group [26 cases]	B group [23 cases]	P-value
Age (mean (±SD))	60.6(±17.3)	57.1(±12.5)	0.423^Ⅰ^
Sex (Man): n (%)	17(65.4)	11(47.8)	0.215^Ⅲ^
Intra-axial tumor, n (%)	21(81)	19(82.6)	0.418^Ⅲ^
Surgical removal, n (%)	20(76.9)	19(82.6)	0.626^Ⅲ^
Preoperative seizure control (number of poor cases (percentile))	4 (15.4%)	5 (21.7%)	0.716^Ⅲ^
AST: median (95% CI)	n:26, 19 (5.0)	n:23, 18 (5.5)	0.616^Ⅱ^
ALT: median (95% CI)	n:26, 15.5 (8.7)	n:23, 18 (9.6)	0.748^Ⅱ^
LDH: median (95% CI)	n:19, 167 (11.7)	n:19, 162 (15.8)	0.773^Ⅱ^
Cre: median (95% CI)	n:26, 0.73 (0.13)	n:23, 0.62 (0.07)	0.067^Ⅱ^
eGFR: median (95% CI)	n:26, 78.2 (8.7)	n:23, 89.2 (7.4)	0.161^Ⅱ^

Among them, there were 17 patients who had been taking AEDs with a provisional diagnosis of epilepsy without seizures in the past. Of all 49 patients, 40 (81.6%) had intra-axial tumors, including 26 glioblastomas (GBMs), five WHO Grade III gliomas (three anaplastic astrocytoma, two Anaplastic oligodendroglioma), eight low-grade gliomas (four oligodendroglioma, two diffuse astrocytoma, one pilocytic astrocytoma, one oligoastrocytoma), and one metastatic tumor. Of these, there were 21 frontal, 10 temporal, five parietal, one temporoparietal, one occipital, one basal ganglia, and one multiple lesions. Nine patients had extra-axial tumors, including eight meningiomas and one inflammatory lesion. Thirty-two out of them had an obvious history of epileptic seizures. As for postoperative epileptic seizures, they occurred only in three out of 49 patients during the first week (6.1%) and in seven patients within two weeks after surgery (14.3%, including three cases during the first week). Six (85.7%) out of the seven patients with seizures had intra-axial gliomas, although there was no significant difference between them and 42 patients without epileptic seizures, as 34 (76.5%) of those 42 also had intra-axial gliomas (p>0.1, Fisher direct method). Six out of the seven epileptic patients had an obvious history of seizures. All epileptic cases were under good control and in six (85.7%) out of the seven cases lesions were located on the left side. The mean age of the seven patients was 67.6 years and four of them were men.

In Group A, epileptic seizures occurred in two out of 26 patients during the first week (7.7%) and in five patients during the first two weeks after surgery (19.2%). In Group B, seizures occurred in one out of 23 patients (seven patients in Group B1 and 16 patients in Group B2) during the first week (4.3%) and in two patients during two weeks (8.7%) after surgery. Lower complication grade of epileptic seizures was observed in Group B (CDC grade IV in three cases and CDC grade II in two cases), rather than in Group A (CDC grade II in two cases), although no significant difference was noted. There was no significant difference in the total number of seizures, NCSE, NCSE/GTCS, or focal seizures (Tables 2, 3).

**Table 2:** Postoperative seizures during the first two weeks in Group A and Group B CDC, The Clavien-Dindo Classification; FAS, focal awareness seizures; FIAS, focal impaired awareness seizures; GTCS, generalized tonic-clonic seizures; NCSE, non-convulsive status epilepticus *2 cases of postoperative seizures during the 1st week. **1 case of postoperative seizure during the 1st week. II, Fisher’s exact test.

	Group A (26 cases)	Group B (23 cases)		P-value^Ⅱ^
		Group B1 (7 cases)	Group B2 (16 cases)	
GTCS	1/26 (3.8%)	0/7	0/16	
NCSE	2/26 (7.7%)	0/7	0/16	
NCSE / GTCS	3 (11.5%)	0/23 (0.0%)		0.141
FIAS	1/26 (3.8%)	0/7	0/16	
FAS	1/26 (3.8%)	0/7	2/16 (12.5%)	
Focal seizures	2/26 (7.7%)	2/23 (8.70%)		0.647
CDC grading	IV (3 cases) and II (2 cases)	II (2 cases)		0.256
Total seizures	5*/26 (19.2%)	2**/23 (8.70%)		0.424

**Table 3:** Detailed data of patients having postoperative seizures AA, Anaplastic astrocytoma; AED, anti-epileptic drug; CBZ, carbamazepine; CDC, The Clavien-Dindo Classification; Front, frontal lobe; FAS, focal awareness seizure; FIAS, focal impaired awareness seizure; GBM, glioblastoma; GTCS, generalized tonic-clonic seizure; IDH, isocitrate dehydrogenase; LEV, Levetiracetam; LGG, low-grade glioma; LV, Lateral ventricle; NCSE, non-convulsive status epilepticus; Occi, occipital lobe; OD, Oligodendroglioma; Parie, parietal lobe; PA, pilocytic astrocytoma; POD, postoperative days

Age/Sex	Diagnosis	Side	Type of surgery	Group	Obvious prior seizure	Preoperative AEDs (mg/day)	Preoperative seizure control	Postoperative seizure type and CDC grade	Postoperative seizure onset
67 /F	IDH-wildtype GBM recurrence	Left Occi.	Removal	A	Yes	LEV 1000mg	Good	NCSE (Grade IVa)	8 POD
84/M	IDH-wildtype AA	Left Pariet.	Biposy	A	No	LEV 1000mg	Good	NSCE (Grade IVa)	9 POD
82/M	IDH-wildtype GBM	Left Front.	Biopsy	A	Yes	LEV 1000mg	Good	FIAS (Grade II)	3 POD
69/M	Fibrous meningioma	Right LV	Removal	A	Yes	LEV 2000mg ZNS 200mg CBZ200mg	Good	GTCS (Grade IVa)	3 POD
70/M	IDH-wildtype GBM	Left Front.	Biopsy	B2	Yes	LEV 1000mg	Good	FAS (Grade II)	11 POD
46/F	IDH-mutant OD	Left Front.	Removal	B2	Yes	LEV 1000mg	Good	FAS (Grade II)	5 POD
55/F	Unclassified LGG (PA suspected)	Left Pariet.	Removal	A	Yes	LEV 1000mg CBZ 600mg	Good	FIAS (Grade II)	11 POD

For patients with NCSE, electroencephalogram (EEG) was performed and rhythmical epileptiform discharges were observed on one patient, while the diagnosis was made based on clinical findings without EEG on the other one.

Adverse effects associated with liver, kidneys, and other adverse events determined on postoperative one and two weeks in the three groups are shown in Table 4.

**Table 4:** Adverse effects associated with liver, kidneys, and other adverse events determined on postoperative days (POD) 7 (one week) and 14 (two weeks). AE, adverse events; ALT, alanine aminotransaminase; AST, aspartate aminotransaminase; CDC, The Clavien-Dindo Classification; Cre, creatinine; G, CTCAE Grade; eGFR, estimated glomerular filtration rate * Possible association with AEDs, ** Unlikely association with an AED

		Group A (26 cases)	Group B1 (7 cases)	Group B2 (16 cases)
POD7	AST	None	None	None
	ALT	G1 in 2 cases* G2 in 1 case*	None	G1 in 1 case*
	Cre	G2 in 1 case**	None	None
	eGFR	G2 in 2 cases**	None	None
	Other AEs excepting above data or epileptic seizure (until POD7±2)	None	G2 Depression in 1 case**	None
POD14	AST	None	None	None
	ALT	None	None	None
	Cre	None	None	None
	eGFR	G2 in 2 cases**	None	G2 in 1 case**
	Other AEs excepting above data or epileptic seizure (POD8±2 to POD 14±2)	None	None	None

**Figure 1:**
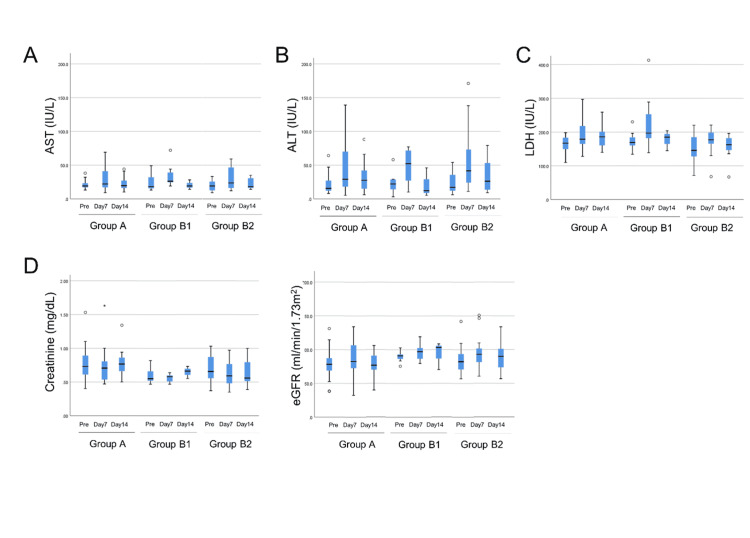
Blood test data before surgery, seven days after surgery and 14 days after surgery. All data are shown as box plots. ALT: alanine amino-transaminase; AST: aspartate amino-transaminase; LDH: lactate dehydrogenase; eGFR: estimated glomerular filtration rate

There was no difference in the frequency of adverse effects in each group. Figure 1 shows box plots of blood test data before surgery, one week after surgery, and two weeks after surgery. No significant difference in blood test data associated with hepatic damage, kidney function, or other organ damage was seen among the three groups both during one and two weeks after surgery (p>0.1, Mann-Whitney U test).

## Discussion

History of epileptic seizures, gliomas (especially LGGs), and extent of tumor resection (lesser than gross total one) predispose patients to early seizures [5, 17, 18]. Iuchi et al. reported that epileptic seizures during the first week after surgery occurred in 9.1% of glioma patients, where PHT was given mostly for prophylaxis [5]. Etiology is not fully known, elevated levels of glutamate, induced by glioma, can lead to hyperexcitability and seizures [19, 20].

In this study, epileptic seizures within one week after surgery occurred in three out of 49 patients (6.1%) in total, 7.7% in the LEV alone group and 4.3% in the LEV+SCBs group, which was consistent with our previous report showing 5% incidence in brain tumor patients using LEV, but it was relatively frequent compared to other studies that showed only 1.9%-2.5% incidence when using LEV [8, 21]. Such a high frequency may be owing to proactive confirmation of epileptic seizures including NSCE. Also, all 49 patients in the current study had been taking AEDs, and 65.3% of them had a history of obvious seizures; all the patients with early seizures during the first week and 85.7% of patients with seizures during the first two weeks after surgery had a history of obvious seizures. As for the tumor type, 79.6% of the patients in this study had gliomas, which can contribute to a higher seizure incidence [5] [17, 18]. There might be a difference by focusing on patients with intra-axial tumors rather than all patients with brain tumors. Although there was no significant difference in seizures, LEV plus SCBs treatment resulted in fewer and milder seizures, and there were no cases of status epilepticus in this group. There was no significant difference in other adverse events including hepatic and kidney function/damage marker-associated parameters between the two groups and no other severe adverse effects were observed in the two groups, suggesting that additional use of SCBs after brain tumor surgery is safe and doesn’t induce any additional adverse events. Since the safety of the add-on was shown in this study, we are planning another clinical trial to determine whether the add-on is effective in certain populations, in a larger number of brain tumor patients in several facilities and focusing on patients’ characteristics, such as the presence of gliomas and the history of obvious seizures.

Limitations included conducting a single-center study, the number of patients, and the inclusion of patients who received fPHT instead of intravenous LCM at the early stage of the study. Additionally, although all patients in the study had a preoperative diagnosis of epilepsy, some patients were provisionally diagnosed with epilepsy by doctors in referral hospitals and started receiving prophylactic medications without obvious prior seizures.

## Conclusions

In conclusion, the incidence of epileptic seizures during one and two weeks after surgery was 7.7% and 19.2% in the LEV alone group and 4.3% and 8.7% in the LEV+SCBs group, respectively, without significant difference, but there were fewer and milder seizures and no cases of major seizures, such as NCSE or GTCS, in the combination treatment group. There was no hepatic damage or renal function worsening with the addition of SCBs in the combination group, suggesting that such treatment could be done safely.
